# Skin dysbiosis and loss of microbiome site specificity in critically ill patients

**DOI:** 10.1128/spectrum.03078-23

**Published:** 2024-02-14

**Authors:** Tilman E. Klassert, Cristina Zubiria-Barrera, Luisa Denkel, Robert Neubert, Antony Schneegans, Aylina Kulle, Antje Vester, Frank Bloos, Christian Schulze, Jörg Epstude, Petra Gastmeier, Christine Geffers, Hortense Slevogt

**Affiliations:** 1 Respiratory Infection Dynamics, Helmholtz Centre for Infection Research - HZI Braunschweig, Braunschweig, Germany; 2 Department of Respiratory Medicine and Infectious Diseases, Hannover Medical School, German Center for Lung Research (DZL), BREATH, Hannover, Germany; 3 Institute for Hygiene and Environmental Medicine, Charité - Universitätsmedizin Berlin, Berlin, Germany; 4 ZIK Septomics, Host Septomics, Jena University Hospital, Jena, Germany; 5 Department of Anesthesiology and Intensive Care Medicine, Jena University Hospital, Jena, Germany; 6 Department of Internal Medicine I, Cardiology, Angiology, Intensive Medical Care, University Hospital Jena, Jena, Germany; 7 Department of Hospital Hygiene, Thuringia Clinic "Georgius Agricola", Saalfeld/Saale, Germany; Chengdu University, Chengdu, China

**Keywords:** dysbiosis, skin microbiome, ICU, critical illness

## Abstract

An increasing amount of evidence has linked critical illness with dysbiotic microbiome signatures in different body sites. The disturbance of the indigenous microbiota structures has been further associated with disease severity and outcome and has been suggested to pose an additional risk for complications in intensive care units (ICUs), including hospital-acquired infections. A better understanding of the microbial dysbiosis in critical illness might thus help to develop strategies for the prevention of such complications. While most of the studies addressing microbiome changes in ICU patients have focused on the gut, the lung, or the oral cavity, little is known about the microbial communities on the skin of ICU patients. Since the skin is the outermost organ and the first immune barrier against pathogens, its microbiome might play an important role in the risk management for critically ill patients. This observational study characterizes the skin microbiome in ICU patients covering five different body sites at the time of admission. Our results show a profound dysbiosis on the skin of critically ill patients, which is characterized by a loss of site specificity and an overrepresentation of gut bacteria on all skin sites when compared to a healthy group. This study opens a new avenue for further investigations on the effect of skin dysbiosis in the ICU setting and points out the need of strategies for the management of dysbiosis in critically ill patients.

**IMPORTANCE:**

Unbalanced gut microbiota in critically ill patients has been associated with poor outcome and complications during the intensive care unit (ICU) stay. Whether the disturbance of the microbial communities in these patients is extensive for other body sites, such as the skin, is largely unknown. The skin not only is the largest organ of the body but also serves as the first immune barrier against potential pathogens. This study characterized the skin microbiota on five different body sites in ICU patients at the time of admission. The observed disturbance of the bacterial communities might help to develop new strategies in the risk management of critically ill patients.

## OBSERVATION

The disruption of the bacterial structures in the gut microbiota, defined as dysbiosis, has been increasingly reported in intensive care unit (ICU) patients and linked to a variety of conditions such as major burns, acute respiratory distress syndrome, and sepsis (1–5). Several studies have thereby reported a significant association between gut dysbiosis and disease severity and mortality in critically ill patients (3, 6, 7). In addition, a dysbiotic status of the gut microbiome has been suggested to contribute to an increased vulnerability of ICU patients to complications such as nosocomial infections and malfunctioning of immune processes (8–10). A better understanding of the microbial dysbiosis in critical illness might thus help to develop strategies for the prevention of such complications.

In recent years, our understanding of dysbiosis in critical illness and its impact on clinical outcomes has expanded beyond the gut microbiota. A few studies have already reported a dysbiotic status of both the oral (8) and the pulmonary (6, 11) microbial communities in ICU patients. However, microbiome changes in other body sites, such as the skin, remain largely unexplored in the context of critical illness. While a few studies have already pointed toward a loss of bacterial diversity (12, 13) and a shift in dominant taxa (14) on certain skin sites during ICU stay, a comprehensive study of the different bacterial communities that populate the skin of these patients is still to be performed.

On the skin, specific microbial communities can be found on different body sites, as the physiological characteristics of each niche (sebaceous, oily, or dry) shape the composition of the microbial community structures (15). At each of these sites, the skin microbiome plays crucial roles in the breakdown of natural products, the defense against external pathogens, and the training of our immune system (15). Thus, changes in the skin microbiome at different skin sites could play an important role in the risk management of ICU patients. This observational study aimed to characterize the skin microbiome in ICU patients at five different body sites (axillary vault, gluteal crease, hypothenar palm, nares, and external auditory canal) in comparison to a healthy cohort. A total of 265 samples from 26 ICU patients and 27 healthy volunteers were included in this study and subjected to 16S rRNA gene sequencing approaches (see detailed methods and study design in the Supplementary Material and Fig. S1).

## RESULTS

The sequencing of the skin samples of both the healthy volunteers and the ICU patients at the time of admission retrieved mainly bacteria belonging to the genera *Corynebacterium*, *Staphylococcus*, and other Gram-positive cocci (such as *Anaerococcus*, *Finegoldia*, or S*treptococcus*), which are frequently found in skin samples (Fig. S2). *Staphylococcus* was the most dominant genus overall (with a relative abundance range of 15.7–55.6%), while *Corynebacterium* was highly present (>16.7% relative abundance) in the axilla, the gluteal crease, and the nare samples. *Anaerococcus* spp. were best represented in the samples from the axilla and the gluteal crease; *Streptococcus* spp., on the hand palm; and *Finegoldia* spp., in the gluteal crease. The ear samples were rather characterized by a high presence of typical otic taxa, such as *Alloiococcus* or *Turicella*. The nare samples showed relatively high presence of *Dolosigranulum* (in both cohorts) and *Lawsonella* (in the healthy group; see Fig. S2).

**Fig 1:**
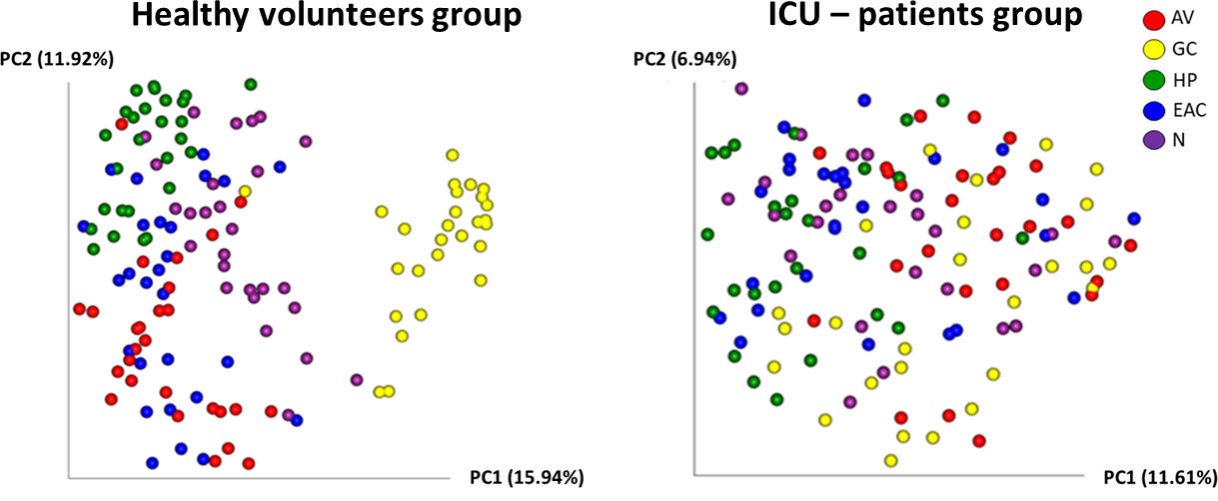
Loss of microbiome site specificity in ICU patients. Principal coordinate analysis of the β-diversity of the skin microbiome (all sites) using unweighted Unifrac distances. Shown are the distances and clustering patterns between different sampling sites (AV = axillary vault; GC = gluteal crease; HP = hypothenar palm; EAC = external auditory canal; *N* = nares) for each of the study cohorts (healthy volunteers and ICU patients).

When exploring the diversity metrics in our data set, α-diversity analysis only retrieved significant differences between cohorts on the hand palms (Fig. S3A). Principal coordinate analyses (PCoA) of the β-diversity showed highly significant (*P* < 0.001; Permanova) segregation between the microbial populations of both cohorts at all skin sites (Fig. S3). Since the effect sizes and clustering effects between groups were strong (pseudo F values ranging between 2.2 and 5.0), further diversity metrics were performed to analyze the inter-site distances in each of the cohorts. The integrative analysis of the whole skin microbiome (all five sites in one PCoA space) revealed a site-specific clustering pattern in the healthy group, as all pairwise site comparisons showed significant differences between each other (*P* < 0.05; Permanova). Interestingly, this site-dependent segregation was not observed in the ICU patients (Fig. 1), suggesting a loss of site specificity in this cohort. Permanova tests confirmed a loss of significance (*P* > 0.05) among different pairwise site comparisons (AV/GC and EAC/N) in the data set of the ICU patients.

**Fig 2:**
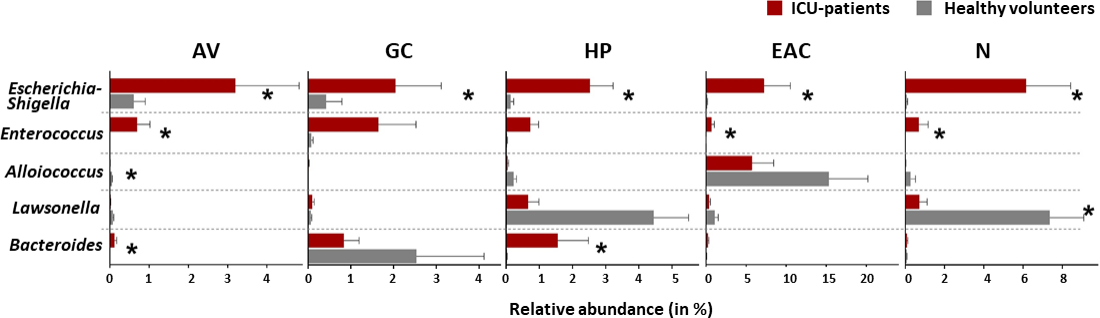
Taxa with differential distribution between study groups. Shown are the relative abundance patterns of the five taxa that showed a differential distribution patterns between cohorts at any of the sampled skin sites (AV = axillary vault; GC = gluteal crease; HP = hypothenar palm; EAC = external auditory canal; *N* = nares). Comparative analyses and significance levels were determined as measured by ANCOM; *W > 100).

The taxonomic data of the two study cohorts were then subjected to ANCOM analysis to resolve the specific taxa with differential distribution profiles between populations (see also Table S3). The results of this analysis revealed a site-overarching increase of typical gut microbes in the ICU cohort, as shown by the increased relative abundances of *Escherichia-Shigella* (all sites) and *Enterococcus* (AV, EAC, and N; see Fig. 2). In addition, we also observed a few site-specific genera with lower relative abundance in the patient group. These included *Alloiococcus* in the axilla and *Lawsonella* in the nares (Fig. 2). The differential relative abundance of the latter one might however be explained by the age difference between our study groups, since the presence of this particular genus on the skin has been shown to be age dependent (16, 17).

The rapid loss of microbial balance observed across all different skin samples from ICU patients in this study is in line with the findings reported by different groups on other skin sites (12, 14). However, comparative analysis of the compositional data of these studies, including ours, also shows a certain variance in the taxa that are reported as dominant across different skin sites. One plausible explanation might be the choice of different amplicon regions for taxonomic assignment. Thus, study designs that rely on the V4 region, like ours, might result in an underrepresentation of taxa such as *Propionibacterium acnes* (18).

The disturbance of the skin microbiome observed in our ICU cohort was characterized by a loss of site specificity of the microbial communities from different skin sites. A similar effect has been already described in critically ill by Lamarche et al. (6), who reported a loss of biogeographical distinction between the gastrointestinal tract and the lower respiratory tract (6). Our data suggest that the loss of site specificity on the skin might be associated with the ubiquitous presence of enteric bacteria (such as *Escherichia* and *Enterococcus*). In line with our findings, Bansal et al. (19) already reported *Enterobacteriaceae* to be among the most dominant taxa in nares of critically ill patients (19). These enteric bacteria are not typically found on the skin, and their isolation from skin samples generally correlates with a pathologic status (20). Furthermore, it is notorious that the only bacterial genus with significantly increased presence on all different skin sites in the ICU cohort (*Escherichia*) ranks 4th among the most common nosocomial pathogens in Europe (21).

Interestingly, enrichment with gut bacteria has also been described for the microbiomes of the respiratory tract and the oral cavity in patients with sepsis (4, 8). Furthermore, Dickson et al. (4) could reproduce this phenomenon in septic mouse models. The authors suggested a gut-lung translocation of these enteric bacteria, although the exact mechanisms remain unknown (4). Our study adds to this observation and uncovers the skin as potential reservoir and additional route for the dissemination of enteric bacteria to different body sites in ICU patients (e.g., the lungs of sepsis patients). Beyond systemic infections, this study might also contribute to the investigation of other ICU-related complications, such as pressure ulcers, since a recent study reported the presence of unclassified *Enterococcus* on the skin to have the highest association with pressure ulcer occurrence during hospitalization (22).

This study has some limitations, such as the limited clinical data available for the ICU patients investigated, including their history of antibiotic treatments. Thus, we were not able to associate the microbiome signatures with any therapeutic strategy. However, this study is primarily focused on the comprehensive characterization of the skin microbiota, and the mechanistic influence of antibiotics on the skin dysbiosis might be limited. Indeed, recent studies have shown negligible effects of systemic antibiotics on skin microbiome structures (23–25). Another limitation of this study is the absence of personal hygiene records before admission and the potential hospitalization status of the patients. It is thus unknown to which extent altered hygiene patterns and variable hospitalization lengths of these patients might have differential effects on their bacterial communities. Finally, the limited sample size of this study did only allow for the detection of large effect sizes in the microbiome patterns of the skin in these patients. Smaller effects might remain unnoticed due to the high interpersonal variation observed in the microbial signatures and would thus require larger cohorts to be addressed. Further studies with additional clinical data, larger sample sizes, bacterial viability tests, and a longitudinal design should validate the findings made in this observational study and evaluate routes of modulation for skin dysbiosis in hospitalized patients. Such studies may not only include interventional designs with different topical decolonization strategies but also aim for controlling the effect of the environment and the clinical staff on the skin microbiome. In this sense, a recent study by Li et al. (26) identified the buildings and the human skin to be the main sources of persistence of HAI-related bacteria (including *Escherichia* and *Enterococcus*) detected in the environment of ICUs (26).

In conclusion, the 16S rRNA gene amplicon sequencing analyses of the skin microbiome in ICU patients revealed a profound dysbiosis at different skin sites at the time of patient admission. The results of this study open a new avenue for further investigations on the effect of skin dysbiosis on ICU outcomes while pointing out the need for interventional studies and novel strategies for monitoring and modulating the skin microbiome in the ICU setting.

## Data Availability

Detailed methods are available in the supplemental material. The data sets generated in this study are available at the SRA database under Bioproject accession number PRJNA909975.

## SUPPLEMENTAL MATERIAL

Supplemental material spectrum.03078-23-s0001.pdf Supplemental methods, Fig. S1 to S3, and Tables S1 to S3.

ASM does not own the copyrights to Supplemental Material that may be linked to, or accessed through, an article. The authors have granted ASM a non-exclusive, world-wide license to publish the Supplemental Material files. Please contact the corresponding author directly for reuse.
