# Supplementary material for: Skin dysbiosis and loss of microbiome site specificity in critically ill patients
Source: Microbiol Spectr. 2024 Feb 14;12(3):e03078-23. doi: 10.1128/spectrum.03078-23 (PMC10913461; doi:10.1128/spectrum.03078-23)
Supplement: Supplemental material — Supplemental methods, Fig. S1 to S3, and Tables S1 to S3. [file spectrum.03078-23-s0001.pdf]

SUPPLEMENTARY MATERIAL (DETAILED METHODS)

Study design and sampling

This observational study aimed to characterize the skin microbiome in ICU-patients covering five different body sites: the axillary vault (AV), the gluteal crease (GC), the hypothenar palm (HP), the nares (N) and the external auditory canal (EAC). These sites were selected in order to obtain a good representation of different microbial communities in dry, moist and sebaceous microenvironments. An overview of the study design is provided in Suppl. Fig. S1. Twenty-six ICU-patients (age range 25-90y; η = 62.5y) from 3 different hospitals (7 patients from Charité – Universitätsmedizin Berlin, 9 patients from Jena University Hospital, and 10 patients from the Thüringen-Kliniken Gregorius Agricola in Saalfeld). Any patient with an anticipated ICU-stay of more than 5 days was eligible for this study and sampling was performed by trained personnel within the first 12 hours after ICU admission. Twenty-seven healthy volunteers (age range 20-61y; η = 35 y) not receiving antimicrobial therapy within the last 6 months served as a control group. Informed consent was obtained for all participating individuals in accordance with the Declaration of Helsinki and the Ethics Committees of Charité – Universitätsmedizin Berlin (ID: EA1/1093/16). This study was conducted within a cluster randomized controlled trial (cRCT) that was registered at the German Register for Clinical Studies (Deutsches Register für Klinische Studien, DRKS00010475). In all cases, sampling was performed by swabbing. The sterile swabs used for skin sampling were premoistened in PBS buffer before the samples were taken. After sampling, each swab was stubbed out in a labeled 1.5 ml Eppendorf-tube prefilled with 300 µl PBS, and immediately stored at -80°C until further use. The PBS tube used for premoistening was used as blank control and subjected to simultaneous sample processing with all other samples from each sampling event.

DNA extraction

DNA isolation was performed using the ZymoBIOMICS DNA Miniprep Kit (Zymo Research) with some additional modifications of the manufacturer's lysis step in order to increase the DNA yield and the coverage of gram positive bacteria. In short: the sample (≈ 250 µl) was transferred to a ZR Bashing-Bead Lysis Tube in addition to 750 µl Lysis Solution and homogenized in SpeedMill Plus (Analytik Jena) using a 5-minute program at maximum speed. Binding and washing steps were performed according to manufacturer's instructions. The elution step was performed with 50 µl of pre-heated (60°C) DNase/RNase free water, and repeated twice.

Library construction and 16S rRNA gene amplicon sequencing

Amplicon libraries and sequencing was performed as described previously (1, 2) and following the guidelines implemented by Caporaso and Walters et al (3, 4). In short. F515-fusion primers with Golay-barcodes and the R806-constructs were used as detailed in Supplementary table S1. The 50 µl PCR reaction was set up on a CAS-1200 pipetting robot (Qiagen) and carried out on a Thermal Cycler S1000 (BioRad) using the Platinum PCR SuperMix (Thermo Fisher Scientific). Thermal conditions included an initial denaturation step (94°C, 3min), 35 amplification cycles (94°C, 15s; 58°C, 20s; 72°C, 30s) and an elongation step at 72°C for 10min. The resulting PCR products were quantified on D1000 Tapes using a TapeStation 2200 (Agilent Technologies), equimolarly pooled and finally purified by size-selection on a 2% SizeSelect E-Gels (Thermo Fisher Scientific). The final libraries were prepared for Illumina sequencing using the MiSeq Reagent Kit v2 (Illumina) following manufacturer's instructions. The sequencing reagents and the run plan were adapted as described by Caporaso and colleagues (3). To account for low diversity read outcomes, the library was spiked with 20% PhiX library (Illumina) and the cluster density kept at 600-800 K/mm^2. Sequencing was performed on an Illumina MiSeq apparatus with 251 cycles.

Sequencing Data Analysis

Raw reads were demultiplexed in *QIIME 2* (5) (https://qiime2.org). After quality assessment the q2-dada2 plugin was used to denoise, filter out chimeric sequences and singletons, join pair-end sequences and dereplicate final high quality sequences (6). For each sample we obtained a minimum of 5000 reads, with a mean sequencing depth of 103692 reads per sample (after passing quality filter). Additional run statistics are detailed in suppl. Table S2. Unless otherwise stated, we used default parameters for all DADA2 functions. Taxonomy assignment of the dada2-output feature table was performed using a pre-trained Naive Bayes classifier. This was trained on the SILVA REF NR 99 database (release 132) and sequences were extracted using the 515F forward (GTGYCAGCMGCCGCGGTAA) and 806R reverse primer (GGACTACNVGGGTWTCTAAT) matching the v4 region of the bacterial 16S rRNA gene (7). Taxonomic classification was performed using the sub-classifying genus option implemented in the SILVA database, which avoids loss of data and allows for differentiation between different bacterial strains within a same genus. Sub-classified genus results in unspecified taxa which is assigned a number. The datasets generated in this study are available at the SRA database under the following Bioproject accession number: PRJNA909975 [https://www.ncbi.nlm.nih.gov/sra/PRJNA909975].

Statistics

Pairwise comparisons of the Shannon Indices was performed using parametric approaches (two-tailed Student's t-test). For beta-diversity metrics, statistical analyses of the dispersion between groups (*betadisper*) was followed by Permanova in order to detect significant clustering patterns between groups. These comparisons were performed pairwise (between the 2 groups for each of the sites (for Suppl. Fig. S3), or between pairs of sites within each group (data from Figure 1)). Dimension reduction for the detection of differentially distributed ASVs between different groups was achieved using ANCOM (8). The significance threshold for all statistical tests was set at $p<0.05$. Statistical analyses and graphic presentations were performed using GraphPad Prism (GraphPad Software, USA).

REFERENCES

1. Klassert TE, Leistner R, Zubiria-Barrera C, Stock M, López M, Neubert R, et al. Bacterial colonization dynamics and antibiotic resistance gene dissemination in the hospital environment after first patient occupancy: a longitudinal metagenetic study. *Microbiome.* 2021;9(1):1-17.
2. Klassert TE, Zubiria-Barrera C, Neubert R, Stock M, Schneegans A, López M, et al. Comparative analysis of surface sanitization protocols on the bacterial community structures in the hospital environment. *Clinical Microbiology and Infection.* 2022.
3. Caporaso JG, Lauber CL, Walters WA, Berg-Lyons D, Huntley J, Fierer N, et al. Ultra-high-throughput microbial community analysis on the Illumina HiSeq and MiSeq platforms. *The ISME journal.* 2012;6(8):1621-4.
4. Walters W, Hyde ER, Berg-Lyons D, Ackermann G, Humphrey G, Parada A, et al. Improved bacterial 16S rRNA gene (V4 and V4-5) and fungal internal transcribed spacer marker gene primers for microbial community surveys. *Msystems.* 2016;1(1):e00009-15.
5. Bolyen E, Rideout JR, Dillon MR, Bokulich NA, Abnet CC, Al-Ghalith GA, et al. Reproducible, interactive, scalable and extensible microbiome data science using QIIME 2. *Nature biotechnology.* 2019;37(8):852-7.
6. Callahan BJ, McMurdie PJ, Rosen MJ, Han AW, Johnson AJA, and Holmes SP. DADA2: High-resolution sample inference from Illumina amplicon data. *Nature methods.* 2016;13(7):581-3.
7. Quast C, Pruesse E, Yilmaz P, Gerken J, Schweer T, Yarza P, et al. The SILVA ribosomal RNA gene database project: improved data processing and web-based tools. *Nucleic acids research.* 2012;41(D1):D590-D6.
8. Mandal S, Van Treuren W, White RA, Eggesbø M, Knight R, and Peddada SD. Analysis of composition of microbiomes: a novel method for studying microbial composition. *Microbial ecology in health and disease.* 2015;26(1):27663.

SUPPLEMENTARY MATERIAL (FIGURES)

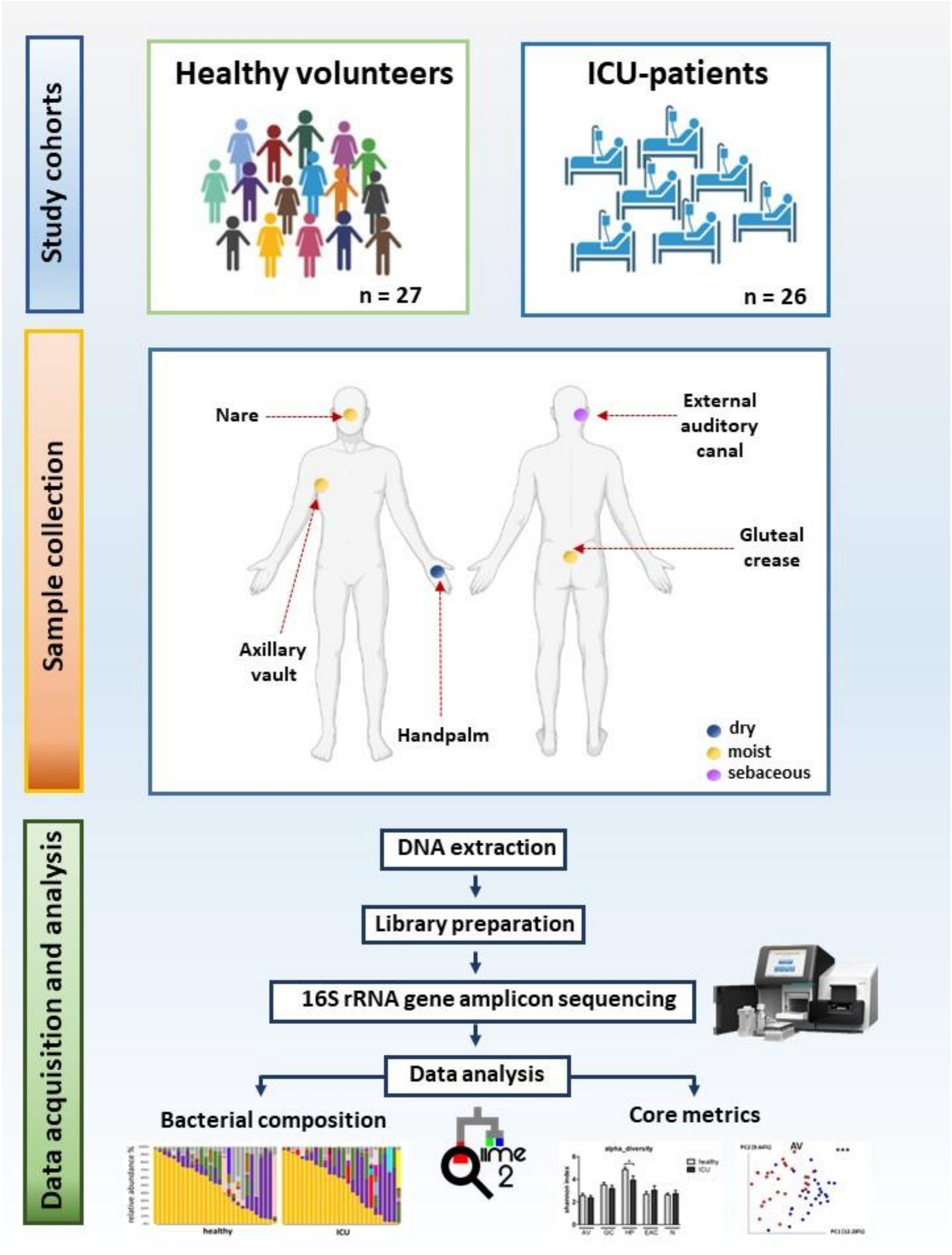

Supplementary Figure S1.- Workflow schematic of the study. The project output included 16S rRNA sequencing data of 5 different skin sites from two cohorts. Data analysis on the bacterial composition of microbial communities on the skin was performed using Illumina sequencing and Qiime analysis tools.

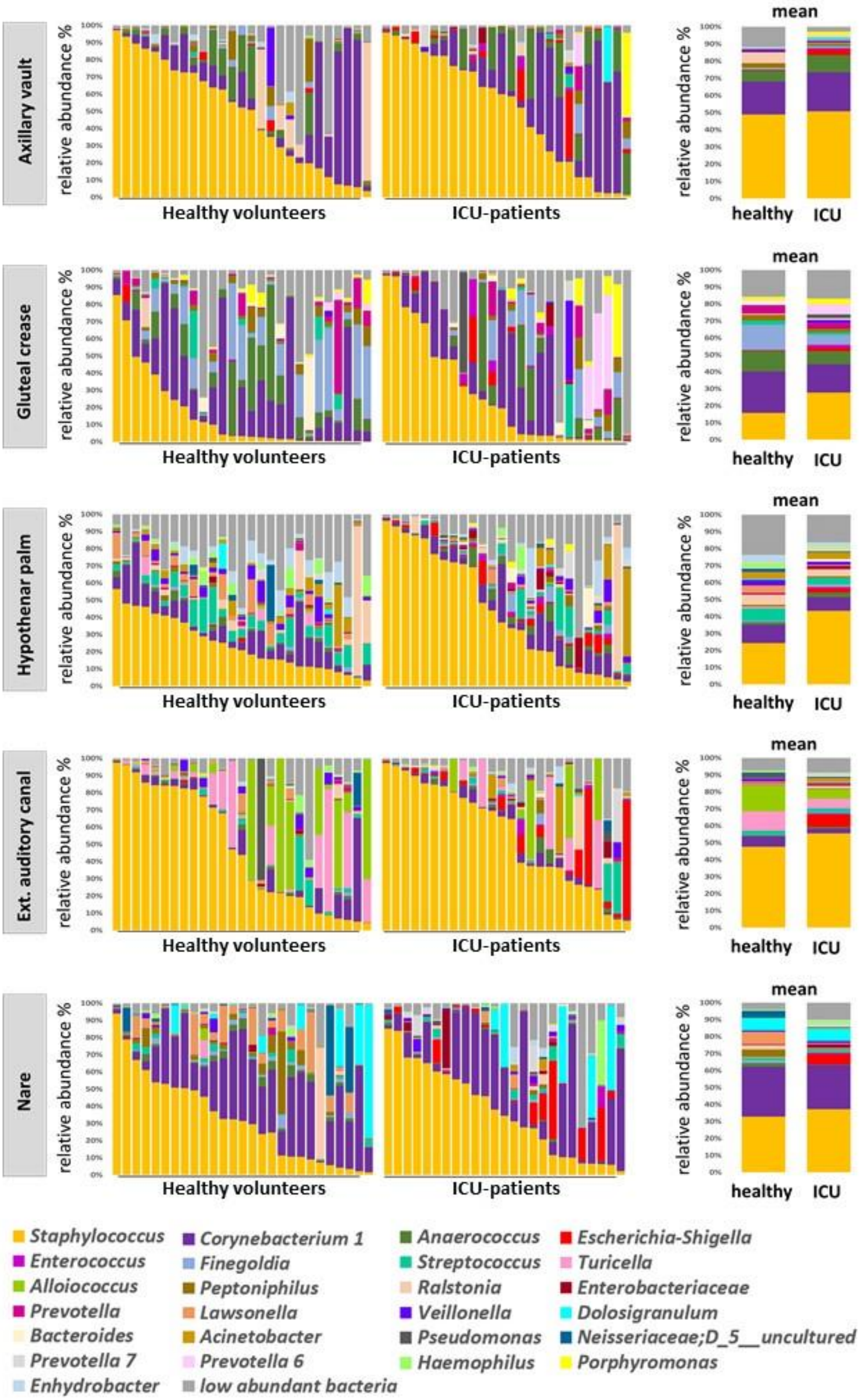

Suppl. Figure S2.- Taxonomic summary with the relative distributions of the microbial communities analyzed separately for each skin site. Shown are the relative abundances of the 25 most important taxa for each individual of the two analyzed cohort (left panel: healthy volunteers vs ICU-patients, ordered by the abundance of the dominant genus), as well as the collapsed mean values for the taxonomies of each group (right panel).

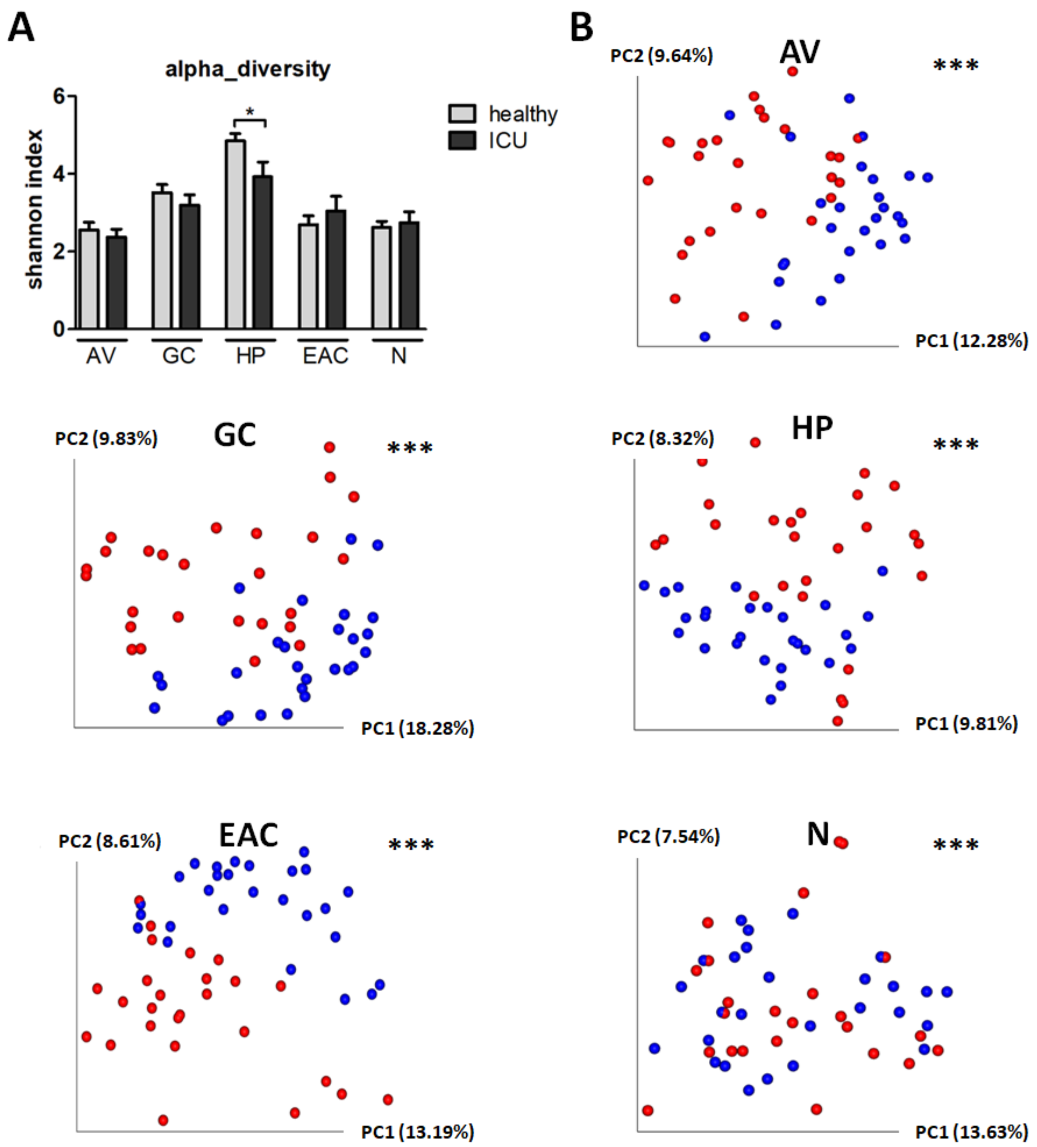

Suppl. Figure S3.- Comparative analysis of the diversity metrics of the skin microbiota between cohorts. **A)** α-diversities of the different skin sites (AV=axillary vault; GC=gluteal crease; HP=hypothenar palm; EAC=external auditory canal; N=nares). Shown are the differences between Shannon indices (mean ± SEM) between cohorts (t-test; *$p < 0.05$). **B)** PCoA of the β-diversity for each site using unweighted Unifrac distances. Statistical significance between healthy subjects (blue) and ICU-patients (red) was calculated by Permanova (***$p < 0.001$).

SUPPLEMENTARY MATERIAL (TABLES)

Supplementary Table S1.- Primer constructs used for the 16S rRNA gene fragment library generation.

	Component	Sequence
**Forward Construct (F515)**	5’ adapter	AATGATACGGCGACCACCGAGATCTACACGCT
	Golay barcode	NNNNNNNNNNNN
	Forward primer pad	TATGGTAATT
	Forward linker	GT
	Forward primer (F515)	GTGYCAGCMGCCGCGGTAA
**Reverse Construct (R806)**	**Component**	**Sequence**
	3’ adapter (reverse compl.)	CAAGCAGAAGACGGCATACGAGAT
	Reverse primer pad	AGTCAGCCAG
	Reverse linker	CC
	Reverse primer (R806)	GGACTACNVGGGTWTCTAAT

Supplementary Table S2.- Read statistics for each of the runs made on the MiSeq apparatus. Shown are the input samples obtained for analysis and the reads left after quality filtering and merging paired-end reads.

	input sequences	input passed filter (%)	input merged (%)
run1	106069	91,27	89,44
run2	155130	88,28	86,04
run3	92983	88,10	85,45
run4	92393	90,15	87,10
run5	99856	89,66	87,07
run6	93046	89,99	87,35
run7	97149	81,23	78,66
run8	90495	91,33	89,02
run9	118456	91,18	89,18
run10	147903	83,30	80,45
run11	138967	90,61	89,15
run12	111615	90,86	89,05
**mean**	**112005**	**88,83**	**86,50**

Supplementary Table S3.- Readout and parameters of the ANCOM analysis. Shown are the taxa that were distributed significantly different (W value > 100) between ICU-patients and healthy controls for each of the skin sites (AV=axillary vault; GC=gluteal crease; HP=hypothenar palm; EAC=external auditory canal; N=nares), indicating the W-value, the feature counts (FC) and the relative abundance (RA) obtained for each taxa.

	**Taxa**	**ANCOM W**	**FC_healthy**	**FC_ICU**	**RA %_healthy**	**RA %_ICU**
**AV**	*Enterococcus*	140	1.0	178.75	0.00%	0.67%
	Bacteroides	128	1.0	31.00	0.00%	0.11%
	Alloiococcus	127	58.5	1.00	0.05%	0.01%
	Escherichia-Shigella	121	199.0	1675.75	0.61%	3.08%
**GC**	*Escherichia-Shigella*	143	23.0	450.0	0.42%	2.04%
**HP**	*Escherichia-Shigella*	157	19.0	1973.5	0.14%	2.52%
	Bacteroides	146	23.5	457.5	0.03%	1.56%
**EAC**	*Escherichia-Shigella*	148	59.0	2563.75	0.12%	7.24%
	Enterococcus	147	1.0	404.75	0.01%	0.68%
**N**	*Escherichia-Shigella*	159	15.0	1835.0	0.07%	6.15%
	Enterococcus	156	1.0	197.0	0.00%	0.68%
	Lawsonella	155	9734.0	90.0	7.35%	0.71%
